# The MEK inhibitor selumetinib complements CTLA-4 blockade by reprogramming the tumor immune microenvironment

**DOI:** 10.1186/s40425-017-0268-8

**Published:** 2017-08-15

**Authors:** Edmund Poon, Stefanie Mullins, Amanda Watkins, Geoffrey S. Williams, Jens-Oliver Koopmann, Gianfranco Di Genova, Marie Cumberbatch, Margaret Veldman-Jones, Shaun E. Grosskurth, Vasu Sah, Alwin Schuller, Corrine Reimer, Simon J. Dovedi, Paul D. Smith, Ross Stewart, Robert W. Wilkinson

**Affiliations:** 1 MedImmune Ltd, Granta Park, Cambridge, UK; 2 AstraZeneca Ltd, Cambridge, UK; 3 AstraZeneca Ltd, 35 Gatehouse Lane, Waltham, USA; 4 New address: Immune Insight Ltd, Biohub, Alderley Park, Cheshire, UK; 5 New address: Abbvie Inc, 1 North Waukegan Road, North Chicago, IL 60064 USA

**Keywords:** CTLA-4, MEK inhibitor, Immunotherapy, Microenvironment, Cancer, Myeloid-derived suppressor cells

## Abstract

**Background:**

T-cell checkpoint blockade and MEK inhibitor combinations are under clinical investigation. Despite progress elucidating the immuno-modulatory effects of MEK inhibitors as standalone therapies, the impact of MEK inhibition on the activity of T-cell checkpoint inhibitors remains incompletely understood. Here we sought to characterize the combined effects of MEK inhibition and anti-CTLA-4 mAb (anti-CTLA-4) therapy, examining effects on both T-cells and tumor microenvironment (TME).

**Methods:**

In mice, the effects of MEK inhibition, via selumetinib, and anti-CTLA-4 on immune responses to keyhole limpet haemocyanin (KLH) immunization were monitored using ex vivo functional assays with splenocytes. In a KRAS-mutant CT26 mouse colorectal cancer model, the impact on the tumor microenvironment (TME) and the spleen were evaluated by flow cytometry. The TME was further examined by gene expression and immunohistochemical analyses. The combination and sequencing of selumetinib and anti-CTLA-4 were also evaluated in efficacy studies using the CT26 mouse syngeneic model.

**Results:**

Anti-CTLA-4 enhanced the generation of KLH specific immunity following KLH immunization in vivo; selumetinib was found to reduce, but did not prevent, this enhancement of immune response by anti-CTLA-4 in vivo. In the CT26 mouse model, anti-CTLA-4 treatment led to higher expression levels of the immunosuppressive mediators, Cox-2 and Arg1 in the TME. Combination of anti-CTLA-4 with selumetinib negated this up-regulation of Cox-2 and Arg1, reduced the frequency of CD11^+^ Ly6G^+^ myeloid cells, and led to the accumulation of differentiating monocytes at the Ly6C^+^ MHC^+^ intermediate state in the tumor. We also report that MEK inhibition had limited impact on anti-CTLA-4-mediated increases in T-cell infiltration and T-cell activation in CT26 tumors. Finally, we show that pre-treatment, but not concurrent treatment, with selumetinib enhanced the anti-tumor activity of anti-CTLA-4 in the CT26 model.

**Conclusion:**

These data provide evidence that MEK inhibition can lead to changes in myeloid cells and immunosuppressive factors in the tumor, thus potentially conditioning the TME to facilitate improved response to anti-CTLA-4 treatment. In summary, the use of MEK inhibitors to alter the TME as an approach to enhance the activities of immune checkpoint inhibitors warrants further investigation in clinical trials.

**Electronic supplementary material:**

The online version of this article (doi:10.1186/s40425-017-0268-8) contains supplementary material, which is available to authorized users.

## Background

Antibodies targeting T-cell checkpoint molecules, such as anti-PD-1/PD-L1 and anti-CTLA-4, have been shown to deliver long-term benefits for a subset of cancer patients [1, 2]. One potential way to extend the benefit of checkpoint inhibitors in a broader cohort of patients is through combinations with other classes of anti-cancer therapeutics [3]. However, the choice of combination partner and optimization of dose scheduling for multiple therapies are challenging. These need to be informed by increased understanding of how each therapy affects the immune system and the mechanisms driving combined activity.

Mutations in KRAS that constitutively activate the RAS-RAF-MEK-ERK pathway are commonly found in cancers leading to cell proliferation [4]. Therefore, small molecule inhibitors of MEK have been used in a range of cancer indications and have shown activity [5]. In these cancer settings, patients may benefit from MEK inhibitors used in combination with checkpoint blockade. Taking into account the well-documented role of MEK in T-cell receptor (TCR) signaling [6], there are concerns that MEK inhibition could be detrimental to anti-tumor T-cell responses, and therefore to T-cell mediated immunotherapy approaches. However, recent in vivo studies in mouse models of KRAS-mutant colorectal cancer [7, 8], BRAF^V600E^-mutant melanoma [9], and triple-negative breast cancer [10] demonstrated that the combination of MEK inhibitors and antibodies targeting PD-1 or PD-L1 resulted in superior anti-tumor efficacy compared to single agents. The MEK inhibitor trametinib was also found to synergise with adoptive T-cell transfer [9] and anti-CTLA-4 [8] therapies in mouse tumor models.

A growing body of preclinical evidence describing the immune effects of MEK inhibitors provides several possible explanations for the observed synergy between MEK inhibition and immunotherapy. MEK inhibition has been shown to protect tumor-specific effector T-cells against chronic TCR-driven apoptosis [7], increase the extent of tumor T-cell infiltration [7–9], enhance expression of tumor antigens [8, 9, 11, 12] and MHC class I on tumors [7–10, 13, 14], and reduce the accumulation of monocytic myeloid-derived suppressor cells (mMDSC) in the tumor [15]. However, a comprehensive analysis of the immunological impact of MEK inhibition in the presence of a checkpoint inhibitor, such as anti-CTLA-4 in particular, has not been carried out. Furthermore, the possibility that adaptive resistance mechanisms to checkpoint inhibitor activity can be reversed by MEK inhibition has yet to be explored.

In the present study we sought to characterize the combined effects of MEK inhibition and anti-CTLA-4, and to delineate the effects on the TME driven by the individual treatment arms. Enhanced priming of tumor-specific T-cells is thought to be a principle mode of action for anti-CTLA-4 [16]. Our results demonstrate that the pharmacological inhibition of MEK attenuates, but does not abrogate, anti-CTLA-4-mediated enhancement of immune responses to a foreign antigen. In a mouse model of KRAS-mutant colorectal cancer, anti-CTLA-4 profoundly increases the extent of T-cell activation and infiltration into tumors, and these effects were found to be only minimally impacted by MEK inhibition. Moreover, while treatment with anti-CTLA-4 leads to the induction of immunosuppressive factors such as arginase (*Arg1*) and cyclo-oxygenase-2 (*Cox-2*), we observe these increases can be reversed by treatment with the MEK inhibitor selumetinib. We also demonstrate that sequencing of treatments is an important factor for the optimal combination activity of MEK inhibition and anti-CTLA-4, with sequential but not concurrent treatment resulting in enhanced survival benefit when compared to anti-CTLA-4 treatment alone.

## Methods

### Mice, cell lines and reagents

Experiments used C57BL/6 J or BALB/cAnNCrl mice (Charles River, UK). The CT26 murine colon adenocarcinoma cells (LGC Standards) were maintained in RPMI 1640 media (Gibco) containing 10% fetal bovine serum (FBS). Selumetinib (AZD6244, ARRY-142886) was dissolved in dimethyl sulfoxide (DMSO; Sigma-Aldrich) for in vitro studies; or formulated in 0.5% HPMC +0.1% tween 80 for in vivo studies. Tremelimumab is an anti-human CTLA-4 hIgG2 monoclonal antibody [17]. For in vivo studies a mouse reactive anti-CTLA-4 mIgG2b (clone 9D9) was used (BioXcell).

### In vitro checkpoint inhibitor primary cell assays

Human peripheral blood mononuclear cells (PBMC), isolated from healthy donor leukocones (NHSBT) using Ficoll-Paque Plus (GE Healthcare), were cultured at 2 × 10^5^ cells/well in 96-well plates (Corning) pre-coated with 0.5 μg/mL anti-human CD3 monoclonal antibody (OKT3, eBioscience). Cultures were supplemented with 100 ng/mL staphylococcal enterotoxin A (SEA) (Sigma), 30 μg/mL tremelimumab or isotype control (MedImmune) and selumetinib at the concentrations indicated. Following 72 h incubation at 37 °C, IL-2 release was measured by DELFIA using reagents from a human IL-2 ELISA kit (R&D systems) and europium-labelled streptavidin (Perkin-Elmer). Results were repeated in two independent experiments.

### In vitro phenotypic analysis of antigen presenting cells

Monocytes isolated from PBMCs using human CD14 positive selection kit (Stemcell) were cultured in X-Vivo-15 media (Lonza) with 2% human AB serum (Invitrogen), 1000 IU/mL human GM-CSF and 500 IU/mL human IL-4 (Peprotech) for 6 days. Fresh culture media containing GM-CSF and IL-4 were added on day 3. The resulting monocyte-derived dendritic cells (mDCs) were collected and seeded at 1 × 10^6^ cells/well in 12-well plates. Cells were activated with 0.5 μg/mL of HA-tagged CD40L (R&D systems) and 2 μg/ml of anti-HA antibody (R&D systems); and treated with selumetinib or DMSO control for a further 2 days.

2 × 10^5^ CT26 cells were cultured overnight prior to treatment with selumetinib or DMSO control for 2 days.

For flow cytometry analysis, mDCs were stained with fixable LIVE/DEAD violet (Life Technologies) and incubated with human TruStain FcX (Biolegend) before addition of: CD80-FITC (clone L307.4); CD83-PE (clone HB15e); CD86-APC (clone FUN-1) (BD Biosciences); HLA-DR-PE (clone L243, eBioscience). CT26 cells were stained with a viability stain, H2-Kd-PE (clone SF1–1.1) and PD-L1-APC (clone 10F.9G2, Biolegend). Stained cells were analyzed using a FACScantoII (BD Biosciences).

### Primary immune responses to keyhole limpet hemocyanin (KLH) immunization

Female C57BL/6 J mice were immunized with 300 μg of KLH (Pierce) in Complete Freund’s Adjuvant (CFA, InvivoGen) by subcutaneous injection using 8 mice per group. Selumetinib or vehicle control were administered orally (p.o.) at 25 mg/kg *bis in diem* (*bid*) starting on day 0. Anti-CTLA-4 at 10 mg/kg or saline were administered intraperitoneally (i.p.) at on days 1 and 5. Spleens were harvested on day 8, and 3 × 10^5^ splenocytes were cultured in 96-well plates using DMEM (Invitrogen) containing 10% FBS and 1% penicillin and streptomycin. KLH, or ovalbumin (OVA) (Thermo Scientific) were added to cultures at 100 μg/mL and incubated at 37 °C for 3 days. IFNγ release was measured using a mouse IFNγ assay kit (MesoScale Discovery). Non-specific immune responses were represented by IFNγ production in response to OVA. KLH-specific IFNγ production (pg/mL) was calculated as the level of IFNγ released from cells cultured with KLH (pg/mL) minus the IFNγ released from cells cultured with OVA (pg/mL).

### Tumor studies

BALB/c mice were inoculated s.c. with 5 × 10^5^ CT26 cells. For efficacy studies, mice with measurable tumors were randomized into treatment groups after 4, 7 or 10 days. Selumetinib (25 mg/kg) or vehicle (0.5% HPMC +0.1% tween 80) were administered p.o. twice daily. Anti-CTLA-4 (10 mg/kg) was administered i.p. twice weekly. Tumors were measured three times per week (volume (mm^3^) = length x width^2^/2). Mice were euthanized when tumors reached a volume of 1000 mm^3^. Survival was defined as survival to a humane endpoint, based on tumor volume and overall condition of the animal. For flow cytometry and gene expression analysis studies, tumor-bearing mice were randomized into treatment groups 7 days after cell implantation using the same dose schedules described above. Selumetinib was dosed less than 4 h prior to sample collection for flow cytometric analysis. Flow cytometry and gene expression analyses on CT26 tumors and spleens were performed as described below. Selumetinib was administered 18 h prior to ex vivo cytokine production experiments.

### Flow cytometry

CT26 tumors were digested using a gentleMACS dissociator and a murine tumor dissociation kit (Miltenyi Biotec). Absolute viable cell counts were determined by propidium iodide staining and analysed on the MACSQuant analyzer. Cells from CT26 tumors and splenocytes were stained with fixable LIVE/DEAD blue (Life Technologies) and incubated with anti-mouse CD16/CD32 (eBioscience) prior to addition of anti-mouse: CD8-Pe-Cy7 (clone 53–6.7); CD3-eFluor 450 (clone 17A2); CD11c-PE (clone N418); CD86-FITC (clone GL1); PDCA1-APC (clone 129c) (eBioscience); PD-L1-BV421 (clone 10F.9G2); I-A/I-E (MHCII) (clone M5/114.15.2); B220-BV605 (clone RA3-6B2) (Biolegend); CD45-BV785 (clone 30F11); CD4-BUV395 (clone GK1.5); CD11b-BUV395 (clone M1/70); Ly6G-APC-Cy7 (clone 1A8); Ly6C-PerCP-Cy5.5 (clone AL-21) (BD Biosciences). For intracellular staining, cells were permeabilized using Foxp3 / Transcription Factor Staining Buffer Set (eBioscience) and incubated with anti-mouse Foxp3-PE (clone FJK-16S) and Ki67-eFluor 660 (clone SolA15) (eBioscience). Stained cells were fixed in 3.7% formaldehyde and analyzed using a BD LSRFortessa (BD Bioscience). Data analysis was performed using FlowJo (FlowJo LLC). 8 mice per treatment group were included in all flow cytometry analyses.

### Cytokine production by splenic and tumor-infiltrating T-cells

Splenocytes and single-cell suspension of CT26 tumors, from 3 mice per treatment group, were cultured at 1 × 10^6^ cells/well in a 96-well plate pre-coated with 10 μg/mL anti-mouse CD3 (145-2C11, R&D systems) for 5 h in the presence of brefeldin A (eBioscience). Cells were stained with LIVE/DEAD viability stain, incubated with anti-mouse CD16/CD32, followed by anti-mouse CD4-APC-eFluor780 (clone GK1.5), CD8-eFluor450 (clone 53–6.7) and CD45-BV785 (clone 30F11) (eBioscience). Cells were then intracellularly stained with anti-IFNγ-APC (clone XMG1.2, eBioscience) following fixation and permeabilization. Stained cells were analyzed immediately using a BD LSRFortessa.

### Gene expression analysis

Mice with established tumors were treated for 24 h or 8 days (6 mice per treatment group per time-point) with the last dose of selumetinib administered within 4 h prior to tumor sample collection for mRNA extraction. Tumors were disrupted in Buffer RLT using the Tissuelyser II (Qiagen). Total mRNA was isolated and purified from 10 mg of tissue using RNeasy kit (Qiagen), RNA concentration and purity was determined using the Nanodrop 8000 (Thermo Scientific).

For gene expression analysis using the nCounter Analysis System (Nanostring Technologies), 100 ng of total mRNA were analyzed using the Mouse Immunology Panel (547 genes) (Nanostring Technologies) following manufacturer’s instructions. Data was normalized and log2 transformed through Pipeline Pilot Tool (NAPPA http://CRAN.R-project.org/package=NAPPA). A transcript was designated as not detected if the raw count was below the average of the 8 internal negative control raw counts plus two standard deviations. Differential gene expression analysis was carried out using a multiple testing corrected t-test according to Benjamini and Hochberg.

For quantitative reverse transcription PCR (qRT-PCR), mRNA was reverse-transcribed into complementary DNA (cDNA) using the High Capacity cDNA Reverse Transcription Kit (Life Technologies). 500 ng of cDNA pre-mixed with TaqMan Fast Advanced Master Mix was added to Custom TaqMan Array 384-well Cards (see Additional file 1: Table S2, Life Technologies). qPCR was performed using the QuantStudio 7-flex Real-Time PCR system (Life Technologies) with recommended settings. Expression levels were normalized against Act, Gusb and Hprt1 genes.

### Immunohistochemical (IHC) analysis

Formalin-fixed paraffin embedded (FFPE) tissues were cut to 4 μm sections and IHC was performed using Lab Vision Autostainer-720 (Thermo Scientific) as follows: 3% hydrogen peroxide for 10 min, serum-free protein block (Dako X0909) for 20 min, primary antibody (Phospho-p44/42 MAPK (Erk1/2) (Thr202/Tyr204) (20G11) Rabbit mAb, Cell Signaling Technology) diluted 1:50 with Antibody Diluent (Dako) for 1 h, rabbit Envision HRP-linked polymer (Dako) (30 min) and 3,3′-diaminobenzidine (Dako) for 10 min. Counterstaining was conducted using Carazzi’s hematoxylin. No staining was observed in samples incubated with isotype control antibodies. Digital images of stained slides were acquired using an Aperio slide scanner (Leica Biosystems). Slides were annotated manually to exclude areas of poor tissue/staining quality and a positive pixel algorithm was used to analyze positive staining. Thresholds were set for different staining intensities (weak, medium, strong). Results are displayed as percentage pERK positive staining (medium + strong) relative to control group.

In a separate study, formalin-fixed tumors embedded into paraffin blocks were cut to 3 μm sections and IHC was performed using a Ventana Discovery XT Roche. This study contained 6 mice per treatment group. Antibodies used were anti-Arginase −1 (H-52) Rabbit polyclonal antibody (Santa Cruz Biotechnology sc-20,150 Lot#D2414) at a 1:50 dilution, biotinylated anti-rabbit secondary antibody (Vector Labs #PK-6101), DABMap detection kit (Ventana Medical #760–124), Hematoxylin II (Ventana Medical #790–2208) and Bluing Reagent (Ventana Medical #760–2037). The slides were scanned using an Aperio Scanscope XT (Leica) with a 20X objective. For image analysis, two planes of each tumor (100 μm apart) were evaluated; viable areas were manually marked in Aperio Imagescope (version 12.1), and percent positive pixels were quantified using a modified Color Deconvolution algorithm (Aperio version 9).

### Statistical analysis

Unpaired *t*-tests were used to compare between treatment groups for flow cytometry data and cytokine production. Hypothesis testing was two-sided. qRT-PCR data analysis used the comparative C_T_ (∆∆C_T_) method and statistical testing was performed using the ExpressionSuite Software version 1.0.4 (Life Technologies). Log-Rank Mantel-Cox tests were performed on survival data. Groups were considered significantly different when *P* ≤ 0.05.

## Results

### Selumetinib inhibits T-cell activation in response to CTLA-4 blockade in vitro

**Fig. 1:**
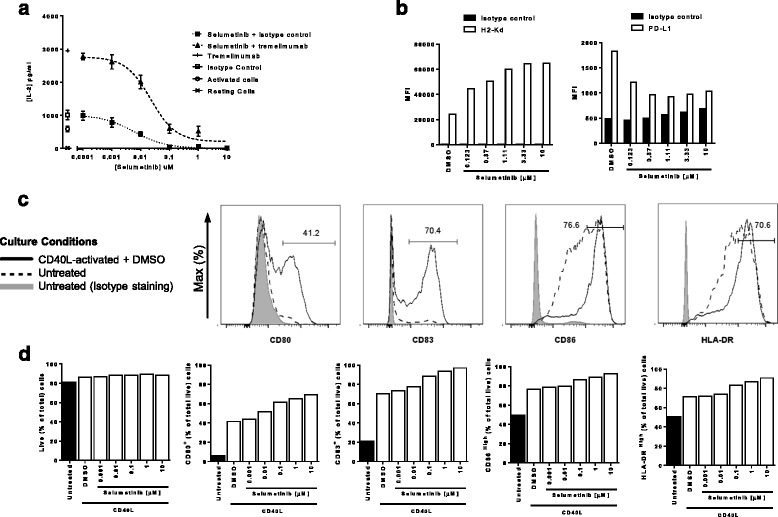
Selumetinib alters the phenotype of antigen presenting cells and suppresses T-cell activation in vitro. **a** Human PBMCs stimulated with SEA, anti-CD3 antibody and either 30 μg/ml of tremelimumab or isotype control, were incubated with increasing concentrations of selumetinib for 72 h. Levels of IL-2 in supernatants were determined by immuno-assay. Data presented as mean (± SEM) of triplicates. **b** Flow cytometry analysis of mouse CT26 tumor cells following 48 h treatment with selumetinib or DMSO vehicle control and stained for H2-Kd and PD-L1. **c** Flow cytometry analysis of human monocyte-derived dendritic cells after 8 days in culture with GM-CSF and IL-4. Cells were either untreated or activated with CD40L and treated with selumetinib or DMSO vehicle control for the last 48 h of culture. Histograms for staining with specific antibodies for mDCs activated with CD40L and treated with DMSO vehicle (solid line); or mDCs left untreated (dashed line); and isotype control staining of untreated mDCs (filled). Percentage of gated cells are shown in histograms for the CD40L-activated + DMSO condition. **d** The percentages of CD80^+^, CD83^+^, CD86^high^ and HLA-DR^high^ cells of total live cells, and frequency of live cells out of total cells are shown. Plotted data are single measurements

Since RAS-MAPK signalling via MEK is downstream of the T-cell receptor [18], we first confirmed the inhibitory effect of selumetinib on T-cell activation. IL-2 was undetectable in unstimulated human PBMCs and increased significantly (*p* = 0.002) upon addition of anti-CD3 and SEA (Fig. 1a). The addition of 30 μg/mL tremelimumab resulted in a 3-fold increase in IL-2 secretion, while an isotype-matched control had no effect (Fig. 1a). Further addition of selumetinib resulted in a concentration-dependent decrease in the level of IL-2 in the presence of both isotype control (IC_50_ = 5.8 nM) or tremelimumab (IC_50_ = 22.3 nM). At concentrations of selumetinib at, or above 1 μM, the release of IL-2 in cultures containing tremelimumab were no different to those containing isotype control.

### Selumetinib alters the surface phenotype of tumor cells and dendritic cells in vitro

Next, we investigated whether selumetinib alters the expression of PD-L1 and class-I MHC by tumor cells, as has been described previously for other MEK inhibitors [8, 10, 14]. The expression of H2-Kd class-I MHC proteins on CT26 KRAS^G12D^ mutant mouse colorectal cancer cells was increased, in a concentration-dependent manner following treatment with selumetinib (Fig. 1b). Specifically, H-2Kd expression increased approximately 2-fold vs. vehicle following addition of 0.123 μM selumetinib and approximately 3-fold following addition of 1.11 μM selumetinib. The addition of selumetinib to tumor cells also resulted in a concentration-dependent decrease in the expression of PD-L1 (Fig. 1b), with ~50% down-regulation observed at concentrations of 0.37 μM selumetinib and above.

Inhibition of MEK signalling has also been reported to affect the phenotype of mDCs [6]. To assess the impact of selumetinib on DC phenotype, human mDCs were activated with CD40L, and expression of the activation markers CD80, CD83, CD86 and HLA-DR was assessed by flow cytometry. CD40L increased the proportions of mDCs expressing CD80, CD83, CD86^High^ and/or HLA-DR^High^ (Fig. 1c). The addition of selumetinib resulted in a concentration-dependent further increase in the percentages of mDCs expressing these markers (Fig. 1d). The most notable increases following 1 μM selumetinib were observed for CD80 (41% in vehicle vs. 65%) and for CD83 (70% in vehicle vs. 94%). More modest increases in the proportion of CD86^High^ mDCs were observed (77% in vehicle vs. 89%) and HLA-DR^High^ (71% in vehicle vs. 87%). A similar selumetinib-dependent effect on the expression of activation markers was observed when mDCs were activated with LPS (see Additional file 2: Figure S1).

### Enhanced primary immune response following CTLA-4 blockade is partially suppressed by selumetinib and is associated with the duration of MEK inhibition

**Fig. 2:**
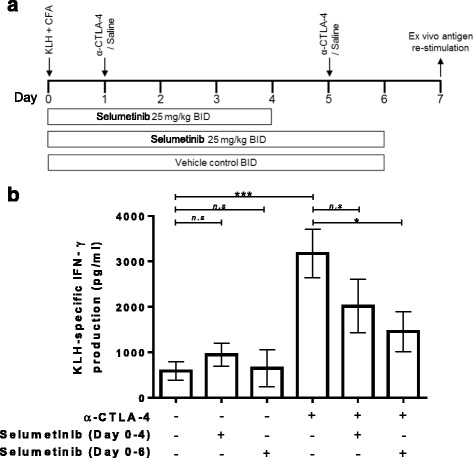
Enhancement of KLH-specific immune response by anti-CTLA-4 is attenuated by continuous combination treatment with selumetinib. **a** Schema showing s.c. injection of keyhole limpet hemocyanin (KLH) in Complete Freund’s Adjuvant (CFA) on day 0.Treatment groups were dosed with either saline/vehicle controls, anti-CTLA-4, or combination of anti-CTLA-4 and selumetinib. Two concurrent dosing regimens were tested for selumetinib and anti-CTLA-4 combination. On day 7, splenocytes were restimulated ex vivo with KLH antigen or ovalbumin (OVA), irrelevant antigen control, for 72 h. **b** KLH-specific IFNγ production by splenocyte cultures are shown (calculated as the IFNγ response to KLH (pg/mL) minus the IFNγ response to OVA (pg/mL)). Data shown are means ± SEM. * *P* < 0.05, ** *P* < 0.01, *** *P* < 0.001 as determined by unpaired *t*-test between indicated groups

Having established that selumetinib up-regulated phenotypic markers of activation on mDCs and inhibited T-cell activation in vitro*,* consistent with previous observations for MEK inhibitors [6], we next explored the overall impact of selumetinib, or selumetinib in combination with anti-mouse CTLA-4, on the generation of primary immune responses to KLH immunization in vivo (Fig. 2a). No sign of toxicity, as determined by piloerection or weight loss, was observed in these studies due to treatment with either anti-mouse CTLA-4 alone or in combination with selumetinib. Therefore this combination was well tolerated.

Ex vivo cultures of splenocytes from KLH-immunized mice produced higher levels of IFNγ in response to KLH protein versus OVA control (see Additional file 2: Figure S2) resulting in 587 ± 204 pg/mL of KLH-specific IFNγ (Fig. 2b). Treatment with selumetinib for 4 days, followed by 3 days off-treatment, resulted in a small increase in the overall KLH-specific IFNγ production to 949 ± 255 pg/mL but did not reach statistical significance. Treatment with selumetinib for 6 days, followed by 18 h off-treatment, resulted in a KLH-specific IFNγ production of 649 ± 407 pg/mL, which was similar to untreated mice.

Anti-CTLA-4 treatment in vivo led to higher levels of KLH-specific IFNγ production (3177 ± 536 pg/mL) compared to immunization alone (Fig. 2b). In comparison to anti-CTLA-4 alone, the combination of selumetinib with anti-CTLA-4 resulted in decreased KLH-specific IFNγ production and the extent of this decrease was greater for animals dosed with selumetinib for 6 days (1458 ± 439 pg/mL) compared to those dosed with selumetinib for 4 days (2016 ± 588 pg/mL).

### Anti-CTLA-4 mediated T-cell responses were unhindered by combining with selumetinib in a CT26 syngeneic mouse tumor

**Fig. 3:**
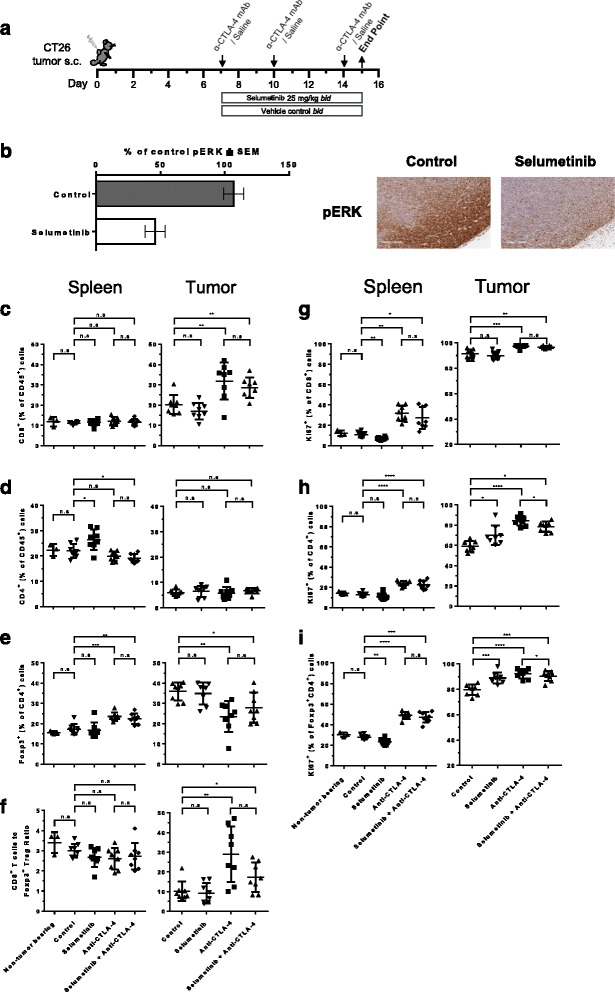
Frequency and effector function of T-cells following selumetinib, anti-CTLA-4 and combination treatment in vivo. **a** Schema showing treatment schedule. **b** Immunohistochemical analysis of tumors for p-ERK 1 h following the last dose with 25 mg/kg selumetinib *bid* (3 doses in total, over 24 h) or vehicle control. Following 8 days of anti-CTLA-4, selumetinib or combination treatment, cells isolated from spleens and tumors were analysed by flow cytometry analysis. Spleens from non-tumor bearing BALB/c mice were also included in the analysis. **c** CD8^+^ T-cell and (**d**) CD4^+^ T-cell populations are presented as percentages of CD45^+^ cells. **e** Frequency of Foxp3^+^ CD4^+^ regulatory T-cells (Tregs) of total CD4^+^ T-cells. **f** Ratio of CD8^+^ T-cells to Tregs. Effects of treatment on the frequency of Ki67-positive cells (**g**) of total CD8^+^ T-cells, (**h**) CD4^+^ T-cells or (**i**) Tregs. Data points in scatter plots represent individual animals, treatment groups each contained 8 mice. Plotted are means ± SD. * *P* < 0.05, ** *P* < 0.01, *** *P* < 0.001, **** *P* < 0.0001, as determined by unpaired *t*-test between indicated groups

To characterize the impact of selumetinib on the immuno-modulatory effects of anti-CTLA-4, we utilized the subcutaneous CT26 mouse colorectal cancer model (Fig. 3a). CT26 cells carry the KRAS^G12D^ mutation [19] and when exposed to selumetinib in vitro demonstrated a concentration dependent decrease in viability (IC_50_ = 1.04 μM, see Additional file 2: Figure S3). Administering selumetinib at 25 mg/kg, twice daily, has previously been shown to be pharmacodynamically active in human tumor xenograft mouse models [20]. In CT26 tumors this dosing schedule also led to a significant decrease in p-ERK levels (Fig. 3b), as demonstrated by immunohistochemical staining.

Profiling of splenic T-cells revealed that selumetinib leads to a small but significant increase in the frequency of CD4^+^ T-cells within all leukocytes, but no change in CD8^+^ T-cells or CD4^+^ Foxp3^+^ regulatory T-cells (Tregs), when compared to control group (Fig. 3c-f, left panel); however, the absolute numbers for all three T-cell populations decreased following selumetinib treatment (see Additional file 2: Figure S4A-C). Within the tumor, selumetinib alone had no effect on T-cell frequencies. In comparison to the control group, anti-CTLA-4 treatment significantly increased the percentage of tumor-infiltrating CD8^+^ T-cells (Fig. 3c), and decreased tumor-infiltrating Tregs (Fig. 3e), with a consequent increased ratio of CD8^+^ T-cells to Tregs (Fig. 3f). Moreover, these intratumoral effects mediated by anti-CTLA-4 were also observed in combination with selumetinib (Fig. 3c-f, right panel).

Although monotherapy with selumetinib appears to have limited impact on the frequencies of T-cells, differential effects on proliferating T-cells were observed within the spleen versus the tumor. In the spleen, selumetinib treatment led to a reduction in proliferating (Ki67^+^) CD8^+^ T-cells and Tregs (as a % of CD45^+^ cells) vs. control group (Fig. 3g, i). In contrast, significant increases in proliferating CD4^+^ T-cells and Tregs (as a % of CD45^+^ cells) vs. control group, were observed in the tumor (Fig. 3h, i). Notably, the overall levels of Ki67^+^ T-cells within the tumor were higher than in the spleen across the groups. In addition, the effects of anti-CTLA-4 were not altered by combining with selumetinib, except for a partial reduction in intratumoral levels of proliferating CD4^+^ T-cells (Fig. 3h). We then explored whether similar changes might occur in effector T-cell function. Selumetinib, whether as monotherapy or in combination with anti-CLTA-4, did not change the percentage of IFNγ-producing T-cells within total splenocytes and tumor samples, following ex-vivo stimulation with anti-CD3, when compared to control or anti-CTLA-4 antibody alone (data not shown). However, this apparent lack of effect may be due to timing of this assessment, therefore time-course studies should be used to further define the effect of selumetinib on T cell effector function in vivo.

### Selumetinib modifies the innate cellular components of the tumor microenvironment

**Fig. 4:**
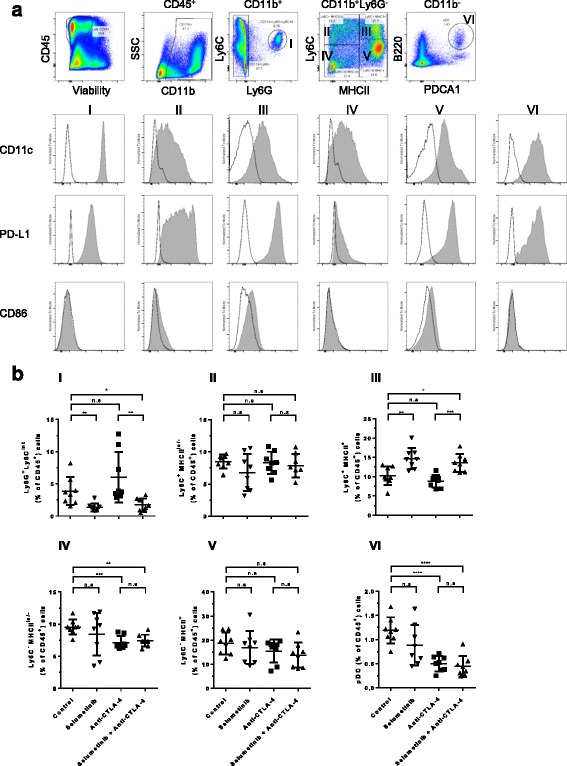
Selumetinib in combination with anti-CTLA-4 alters the composition of innate cells within tumors. On day 8 after initiation of anti-CTLA-4, selumetinib or combination treatment, cells were isolated from tumors and analysed by flow cytometry to identify and characterise myeloid cells and plasmacytoid DCs (pDC). **a** Gating strategy used to identify CD11b^+^Ly6G^+^Ly6C^Int^ (I), CD11b^+^Ly6G^−^Ly6C^+^MHCII^lo/−^ (II), CD11b^+^Ly6G^−^Ly6C^+^MHCII^+^ (III), CD11b^+^Ly6G^−^Ly6C^−^MHCII^lo/−^ (IV), CD11b^+^Ly6G^−^Ly6C^−^MHCII^+^ (V) cells and CD11b^−^B220^+^PDCA1^+^ pDCs. Phenotypic analysis of CD11c, PD-L1 and CD86 expression on cells are shown as histograms with matched isotype staining (black line) and target antigen staining (grey filled). **b** Frequencies of myeloid cells (I-V) and pDCs (VI) out of total CD45^+^ cells. Plotted are mean ± SD. Each group contained 8 mice. * *P* < 0.05, ** *P* < 0.01, *** *P* < 0.001, **** *P* < 0.0001

We next investigated the impact of selumetinib alone, or in combination with anti-CTLA-4, on myeloid cell populations within CT26 tumors following 8 days of treatment (Fig. 3a). CD11b^+^ myeloid cells constituted, on average, >50% of CD45^+^ tumor-infiltrating cells, of which, 5 main populations were identified (I-V) based on expression of MHC-II, Ly6C, Ly6G and CD11c (Fig. 4a); population I phenotypically resembles neutrophil or gMDSC; populations II/III and IV/V encompasses inflammatory monocytes/mMDSC and TAMs, respectively. Furthermore, population III represents the intermediate state in the differentiation of infiltrating monocytes into macrophages at sites of inflammation [21], or into TAMs in cancer [22–24]. Within the CD11b^−^ cell population, plasmacytoid DCs (pDC) (VI) were identified by their surface expression of B220 and PDCA1. Flow cytometry analysis revealed that MHC-II^lo/−^ TAMs (IV), a phenotype associated with proangiogenic activity [22], expressed low or undetectable levels of both PD-L1 and CD86, compared to other populations, which expressed either one or both proteins (Fig. 4a).

Selumetinib monotherapy, and combination with anti-CTLA-4, led to significant decreases in tumor-infiltrating gMDSC/neutrophil when compared to control or anti-CTLA-4 alone (Fig. 4b, panel I); suggesting that MEK inhibition may improve the tumor microenvironment by reducing immunosuppressive cell types. Furthermore, treatment with selumetinib, with or without anti-CTLA-4, increased the frequency of intratumoral MHC-II^+^ Ly6C^+^ intermediary differentiating monocytes (Fig. 4b, panel III). Anti-CTLA-4 alone, or in combination with selumetinib, also led to changes in the myeloid population, seen as a decrease in the frequency of MHCII^lo/−^ TAMs and pDCs (Fig. 4b, panel IV & VI).

### Selumetinib inhibits increases in Cox-2 and Arg1 expression mediated by anti-CTLA-4 therapy without impacting T-cell activation

**Fig. 5:**
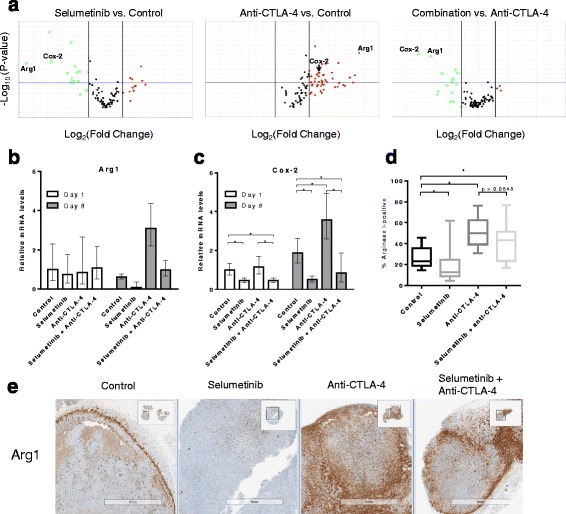
Selumetinib reverses anti-CTLA-4-mediated increases in Cox-2 and Arg1 expression within tumors. Following 8 days of treatment, the relative tumor expression levels of 92 genes were determined by qRT-PCR. **a** Volcano plots showing gene expression levels comparing selumetinib or anti-CTLA-4 treated samples versus control, or combination versus anti-CTLA-4 monotherapy. Down-regulated (green) and up-regulated (red) genes are highlighted. *P-value* boundary of 0.05 and fold-change boundaries of │1.5│ are shown as horizontal and vertical lines, respectively. Bar charts showing tumor expression levels of (**b**) *Arg1* and (**c**) *Cox-2* transcripts, following 1 or 8 days of treatment. Error bars represent 95% confidence intervals. **d** IHC analysis of two planes of tumor (100 μm apart) showing percent positive Arg1. Whiskers shown represents minima and maxima. One-tail Mann-Whitney test performed. * *p* < 0.05 (**e**) Representative Arg1 staining patterns in tumors using IHC. Studies contained 6 mice per treatment group

To provide further context to the flow cytometry data we also characterized expression levels of immune genes in the tumor following treatment. Ninety-two genes (see Additional file 1: Table S2) were evaluated in this study, including genes previously reported to be associated with clinical responses to ipilimumab [2]; 5 candidate genes that we found to be modulated by short-term selumetinib treatment in CT26 tumors using a mouse immunology panel (Nanostring Technologies, see Additional file 1: Table S1); and genes functionally associated with innate and adaptive immunity, immunosuppressive mechanisms and T-cell lineage/subset-associated markers [25]. On day 8, anti-CTLA-4 treatment led to increased expression levels of genes related to T-cell activation (see Additional file 1: Table S4) but also genes related to known immunosuppressive mediators such as *Arg1* [26], when compared to control (Fig. 5a). In agreement with our flow cytometry data, combination treatment did not lead to significant changes in expression levels of genes associated with T-cell activation versus anti-CTLA-4 (see Additional file 1: Table S5B). However, selumetinib treatment reduced baseline tumor expression of *Arg1*, and reverses the increased *Arg1* expression induced in tumor cells by CTLA-4 blockade (Fig. 5a). Interestingly, the effect of selumetinib on *Arg1* expression in the tumor is associated with the down-regulation of *Cox-2* (Fig. 5a-c) on day 8, but not on day 1 when only *Cox-2* is reduced compared to control.

To understand whether the effects on *Arg1* mRNA expression levels translated to the protein level, IHC staining for Arg1 was performed on fixed CT26 tumor sections. Selumetinib treatment alone significantly reduced Arg1-positive staining compared to control (Fig. 5d). However, while combination treatment led to decreased Arg1-positivity compared to anti-CTLA-4 alone, this did not reach statistical significance (*p* = 0.0548, one-tail Mann-Whitney test). Interestingly, we also noted two distinct patterns of Arg1 expression, either strong staining observed in the stromal capsule surrounding the tumor or clustered staining distributed within the tumor (Fig. 5e).

### Pre-treatment with selumetinib enhances the survival benefits of CTLA-4 blockade

**Fig. 6:**
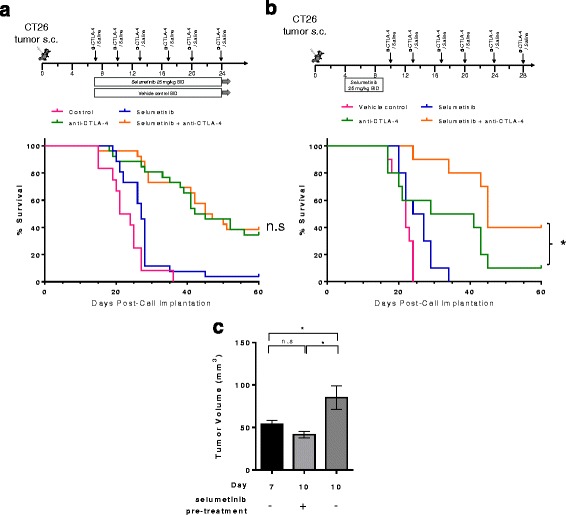
Pre-treatment with selumetinib enhances the activity of anti-CTLA-4 in the CT26 tumor model. **a** Schema showing treatment schedules for vehicle control, selumetinib, anti-CTLA-4 mAb and concurrent combination. Anti-CTLA-4 antibodies were administered on day 7 and then twice per week for a total of 4–6 doses at 10 mg/kg. Selumetinib was administered orally on day 7 at 25 mg/kg twice daily for 18–28 days (**b**) Sequential treatment with selumetinib administered on day 4 for 5 days followed by 1 day of no treatment; anti-CTLA-4 antibody was administered on day 10 and then twice a week for a further 5 doses at 10 mg/kg. Kaplan-Meier survival curves for mice bearing CT26 mouse colorectal tumors are shown. *P < 0.05 as determined by Log-Rank Mantel-Cox test between indicated groups. **c** Tumor volumes (mm^3^) at indicated days post-cell implantation with or without short-term selumetinib pre-treatment (dosing regimen represented in B). Unpaired *t*-tests performed. * *P* < 0.05. Plotted are mean ± SEM. Data shown are representative of 2 independent experiments

Studies to determine whether selumetinib would impact the anti-tumor activity of anti-CTLA-4 treatment were conducted in the CT26 model (Fig. 6a). Median survival in the selumetinib treated cohort was 27 days compared to 22.5 days in the control cohort (*p* < 0.05, Log-rank Mantel-Cox test). When delivered concurrently, combination of selumetinib and anti-CTLA-4 did not provide additional benefit compared to the anti-CTLA-4 treated cohort, but also did not inhibit the effect of anti-CTLA-4.

Having observed both positive effects on the suppressive TME, including reduction of potentially suppressive cell types, and inhibitory effects on the priming of immune responses in vivo following selumetinib treatment*,* we next explored whether a sequential dose schedule, in which animals received selumetinib prior to anti-CTLA-4, could lead to greater antitumor activity. On day 4, mice bearing CT26 tumors were either left untreated or treated with selumetinib for 5 days as indicated (Fig. 6b). Mice pre-treated with selumetinib on day 10 had smaller tumor volumes (41.6 ± 4.0 mm^3^, mean ± SEM) compared to time-matched untreated mice (85.1 ± 13.8 mm^3^, mean ± SEM) (Fig. 6c). Pre-treatment with selumetinib led to significantly longer median survival in the sequential treatment cohort (median survival = 45 days, selumetinib then anti-CTLA-4) compared to anti-CTLA-4 alone (median survival = 35 days, *p*-value = 0.0276). These data indicate that a sequential dosing strategy, in which selumetinib is administered prior to CTLA-4 blockade, leads to enhanced responses in the CT26 tumor model.

## Discussion

Recent studies have shown that MEK inhibition can augment the antitumor T-cell response and synergize with immunotherapy in mice [7–9], however most mechanistic insights were from examining the effects of MEK inhibition alone. We expand on these data by describing the effects of selumetinib, alone or in combination with anti-CTLA-4, on de novo primary immune responses to a model antigen. Subsequently, using the CT26 mouse tumor model we elucidated the effects of selumetinib alone or in combination with anti-CTLA-4 on various T-cell subsets and on the TME. We provide preclinical evidence demonstrating that selumetinib does not significantly impair T-cell mediated anti-tumor activity in the presence or absence of anti-CTLA-4. Furthermore, in the context of CTLA-4 blockade, we reveal beneficial effects of MEK inhibition on the TME. Specifically, selumetinib reduced populations of CD11b^+^ Ly6G^+^ tumor-infiltrating neutrophils or gMDSC, blocked monocytes from differentiating into TAMs at the Ly6C^+^MHCII^+^ intermediate state within the tumor bed [22, 23], and concomitantly inhibited the expression of Cox-2 and Arg-1 – both of which are considered to be key mediators of immunosuppressive pathways [26, 27].

Initially, we hypothesized that MEK inhibition might negate anti-CTLA-4 driven enhancement of T-cell priming in vivo. This idea was based on an appreciation of the role of MAPK signaling during T-cell activation, and on our observation that, selumetinib negated the effect of the CTLA-4 mAb tremelimumab on human PBMCs in vitro. Our data shows that CTLA-4 blockade increases the magnitude of an antigen-specific immune response following KLH immunization. This effect is attenuated, but not abrogated, by concurrent treatment with selumetinib, and appears to be associated with the duration of treatment following antigen challenge. Based on this observation, we clarified that selumetinib limits the expansion phase of antigen-specific immune response but does not prevent initial priming events. This is supported by previous studies showing that adoptively transferred Erk2-deficient OT-1^+^ cells proliferated similarly to wildtype OT-1^+^ cells following OVA immunization, but failed to accumulate in the spleen over time. These studies, together with those showing a role of Erk2, but not Erk1, in the regulation of Bcl-2, Bcl-x and pro-apoptotic Bim expression during CD8^+^ T-cell responses [28], support our hypothesis. In agreement with preclinical reports [8], we demonstrate the complete inhibition of IL-2 production in vitro following T-cell activation in the presence of selumetinib, irrespective of the presence of anti-CTLA-4. In the context of CTLA-4 blockade in vivo, selumetinib may have limited the development of KLH-specific immunity through IL-2-dependent mechanisms during the T cell expansion phase, but not during initial priming. This is consistent with previous studies demonstrating that cell cycle entry and proliferation of recently activated CD8^+^ T-cells is independent of IL-2 [29]. Further work will be required to define the temporal effects of MEK inhibition on naïve CD4^+^ versus CD8^+^ T-cell priming in vivo, which could help inform on the optimal dosing sequence for combining MEK inhibition with checkpoint blockade.

Of particular interest, selumetinib treatment differentially affected T-cells within the spleens of CT26 tumor-bearing animals compared to T cells within the tumors themselves. In spleens, the frequencies of Ki67^+^ CD8^+^ T-cells and Ki67^+^ Foxp3^+^ Tregs were reduced following treatment, while in contrast, frequencies of intratumoral Ki67^+^ CD4^+^ T-cells and Ki67^+^ Foxp3^+^ Tregs were increased. Our data complements findings from *Ebert* et al. [7], suggesting that MEK inhibition may prevent exhausted T-cells from TCR signaling-induced apoptosis and may also enable T cells to proliferate within the tumor. Contrary to previous reports [7, 8], we did not observe increased intratumoral T-cell populations, despite enhanced CD4^+^ T-cell and Treg proliferation. As such, the mechanisms leading to increased T-cell infiltration in response to MEK inhibition, and how this effect correlates with direct antitumor activity, are unclear. Further studies are needed using different preclinical animal models with varying sensitivity to MEK inhibitors, to discern the direct and indirect effects of selumetinib on the host immune response to tumor, as well as time-course experiments to investigate the kinetics of T cell infiltration and activation.

In agreement with others, CTLA-4 blockade led to increased CD8^+^ TILs, depletion of intratumoral Tregs, improved T-cell effector function and generation of a Th1/cytotoxic T-cell response [30]. Importantly, we also showed that these effects persisted in combination with MEK inhibition via selumetinib. However, as with previous studies [7, 8] animals were exposed to MEK inhibition for relatively short periods (less than 12 days). It will be important to determine how T-cell immune responses change over longer-term treatments, which may have clinical implications for dose scheduling and the emergence of resistance.

The presence of MDSCs and tumor-associated macrophages (TAM) has been linked to poor prognosis in cancer patients [31]. These highly heterogeneous and plastic cells are capable of promoting tumor progression, metastasis and suppression of antitumor immune responses via multiple mechanisms [31, 32]. A recent study showed that MEK inhibition reduces accumulation of mMDSC (CD11b^+^ MHCII^−^ Ly6C^hi^ cells) in tumor-bearing mice [15], however, in combination with adoptive cell-therapy, MEK inhibition had no effect on mMDSC levels in tumors of a SM1 mouse melanoma model, but instead, decreased granulocytic-MDSCs (gMDSC, CD11b^+^ Ly6G^+^ Ly6C^+^ cells) [9]. Our data revealed that selumetinib, as a monotherapy, or in combination with anti-CTLA-4, decreased intratumoral CD11b^+^ Ly6G^+^ neutrophil or gMDSC cells in the CT26 mouse tumor model. We also demonstrated for the first time that MEK inhibition by selumetinib resulted in the accumulation of CD11b^+^ Ly6C^+^ MHCII^+^ cells within the TME of tumor-bearing mice; this is a subset of myeloid cells associated with an intermediate state in the differentiation of infiltrating monocytes into macrophages at sites of inflammation [21], or into TAMs in cancer [22–24]. Therefore, MEK inhibition may modify the TME by preventing the accumulation of TAMs through inhibition of monocyte-to-TAM differentiation and polarization.

Correspondingly, we also show that selumetinib led to the down-regulation of immunosuppressive mediators including Cox-2 and Arg1. Others have shown that Arg1 expressing myeloid suppressor cells (i.e. granulocytic- and monocytic-MDSCs, ‘M2-like’ TAMs) can impair antitumor T-cell responses through local L-arginine depletion [26, 27]. Furthermore, recruitment and expansion of myeloid suppressor cells, and the expression of *Arg1* are regulated by the Cox-2/PGE_2_ pathway [33]. A recent study also demonstrated that Cox-2 expression is in part driven by RAF-MEK signaling [34]. Interestingly, we also found that anti-CTLA-4 had the opposite effect, upregulating *Cox-2 and Arg1* transcript levels. The increase in Arg1/Cox-2 following anti-CTLA-4 treatment was negated by combined inhibition of MEK. Taken together, our data demonstrate the ability of MEK inhibition, via selumetinib, to reduce immune suppression in the TME through multiple cellular and molecular mechanisms, and although further studies are required to confirm our observations, suggest that up-regulation of the Cox-2/Arg1 pathway within the tumor could represent an adaptive resistance mechanism to anti-CTLA-4 therapy, which is alleviated through combination with MEK inhibition.

Our preclinical data revealed that a sequential dosing regimen, in which selumetinib was delivered before anti-CTLA-4, led to higher survival rates than either treatment alone. In contrast no additional benefit was observed with concurrent administration of selumetinib and anti-CTLA-4 mAb, compared to CTLA-4 blockade alone. These data highlight dose scheduling as an important factor for optimal therapeutic combination activity. Furthermore, our findings raise the possibility of using MEK inhibitors to not only induce cytoreduction (potentially releasing tumour antigens to prime the immune response), but also to enhance the activity of T-cell checkpoint blockade by augmenting the TME. Although the efficacy of MEK inhibitors as monotherapy or in combination with chemotherapy, in clinical trials have been limited [35], the immune-dependent mechanisms of MEK inhibitors have largely been under-appreciated. Validating the effects of MEK inhibition on the TME in human cancer will be a key step for the successful translation of combination therapy involving MEK inhibitors and immune checkpoint inhibitors. Encouragingly, a phase I clinical trial evaluating sequential therapy with an anti-PD-L1 antibody, durvalumab, following MEK inhibitor treatment has reported evidence of activity in BRAF WT melanoma patients [36]. In addition, the combination of cobimetinib (MEK inhibitor) and an anti-PD-L1 antibody (atezolizumab) is being assessed clinically and has shown promising responses in microsatellite-stable colorectal cancer patients [37]. In summary, these data have informed the translational and clinical strategy for the combination of selumetinib with a checkpoint inhibitor in a phase I study [ClinicalTrials.gov ID: NCT02586987].

## Conclusions

The present study provides a comprehensive characterization of the combined effects of MEK inhibitor and anti-CTLA-4 combination treatment. We demonstrate that pre-treatment with a MEK inhibitor enhances the efficacy of anti-CTLA-4 in a CT26 pre-clinical tumor model. Inhibition of MEK can lead to changes in granulocytic-MDSCs, monocyte-to-TAM differentiation and immunosuppressive mediators Cox-2/Arg1 in the TME, and these changes persist in combination with CTLA-4 blockade treatment. Based on our findings, we propose that MEK inhibition may condition the TME to be more permissive to checkpoint blockade therapy by augmenting the immunosuppressive myeloid compartment. Furthermore, sequencing of MEK inhibitors and anti-CTLA-4, with or without anti-PD-1/PD-L1, will be an important factor to consider in order to optimize their anti-tumor activity.

## Additional files

Additional file 1: **Table S1.** Gene expression analysis of selumetinib treated CT26 tumors (24 h) by Nanostring’s nCounter system. **Table S2.** Target genes selected for qRT-PCR analysis. **Table S3.** Relative tumor gene expression levels post-treatment versus control after 1 day. **Table S4.** Relative tumor gene expression levels post-treatment versus control after 8 days. **Table S5A.** Effect of combination treatment on transcript levels versus anti-CTLA4 (Day 1 post-treatment). **Table S5B.** Effect of combination treatment on transcript levels versus anti-CTLA4 (Day 8 post-treatment). (XLSX 107 kb)

Additional file 2: **Figure S1.** Selumetinib alters the phenotype of LPS-activated human monocyte-derived dendritic cells in vitro. **Figure S2.** Primary immune response to KLH immunization in vivo with or without concurrent selumetinib, anti-CTLA-4 or combination treatment. **Figure S3.** Viability of CT26 mouse tumor cell line exposed to selumetinib in vitro. **Figure S4.** Numbers of splenic and intratumoral T-cell subsets following selumetinib, anti-CTLA-4 and combination treatment in vivo. (PPTX 906 kb)

## Abbreviations

Arg1: Arginase 1
Cox-2: Cyclooxygenase-2, also known as prostaglandin-endoperoxide synthase 2
CTLA-4: Cytotoxic T-lymphocyte-associated protein 4
DC: Dendritic cell
gMDSC: Granulocytic myeloid-derived suppressor cells
IHC: Immunohistochemical
KLH: Keyhole limpet hemocyanin
KRAS: Kirsten rat sarcoma viral oncogene
LPS: Lipopolysaccharide
MAPK: Mitogen-activated protein kinases
MEK: Mitogen-activated protein kinase kinase 1 and/or 2
MHC: Major histocompatibility complex
mMDSC: Monocytic myeloid-derived suppressor cells
OVA: Ovalbumin
PBMC: Peripheral blood mononuclear cells
PD-1: Programmed cell death protein 1
pDC: Plasmacytoid dendritic cells
PD-L1: Programmed death-ligand 1
p-ERK: Phosphorylated extracellular signal-related kinase
PGE_2_: Prostaglandin E2
SEA: Staphylococcal enterotoxin A
TAM: Tumor-associated macrophages
TCR: T-cell receptor
Th1: Type 1 helper
TIL: Tumor-infiltrating lymphocyte
TME: Tumor microenvironment
Tregs: Regulatory T-cells
WT: Wildtype

## References

[1] Daud AI, Wolchok JD, Robert C, Hwu WJ, Weber JS, Ribas A (2016). Programmed death-Ligand 1 expression and response to the anti-programmed death 1 antibody Pembrolizumab in melanoma. J Clin Oncol.

[2] Ji RR, Chasalow SD, Wang L, Hamid O, Schmidt H, Cogswell J (2012). An immune-active tumor microenvironment favors clinical response to ipilimumab. Cancer Immunol Immunother.

[3] Smyth MJ, Ngiow SF, Ribas A, Teng MW (2016). Combination cancer immunotherapies tailored to the tumour microenvironment. Nat Rev Clin Oncol.

[4] Santarpia L, Lippman SM, El-Naggar AK (2012). Targeting the MAPK-RAS-RAF signaling pathway in cancer therapy. Expert Opin Ther Targets.

[5] Caunt CJ, Sale MJ, Smith PD, Cook SJ (2015). MEK1 and MEK2 inhibitors and cancer therapy: the long and winding road. Nature Rev Cancer.

[6] Vella LJ, Pasam A, Dimopoulos N, Andrews M, Knights A, Puaux AL (2014). MEK inhibition, alone or in combination with BRAF inhibition, affects multiple functions of isolated normal human lymphocytes and dendritic cells. Cancer Immunol Res..

[7] Ebert PJ, Cheung J, Yang Y, McNamara E, Hong R, Moskalenko M (2016). MAP Kinase inhibition promotes T cell and anti-tumor activity in combination with PD-L1 checkpoint blockade. Immunity.

[8] Liu L, Mayes PA, Eastman S, Shi H, Yadavilli S, Zhang T (2015). The BRAF and MEK inhibitors Dabrafenib and Trametinib: effects on immune function and in combination with Immunomodulatory antibodies targeting PD-1, PD-L1, and CTLA-4. Clin Cancer Res.

[9] Hu-Lieskovan S, Mok S, Homet Moreno B, Tsoi J, Robert L, Goedert L, et al. Improved antitumor activity of immunotherapy with BRAF and MEK inhibitors in BRAF(V600E) melanoma*.* Sci Transl Med. 2015; 7(279):279ra241. 10.1126/scitranslmed.aaa4691 PMC4765379 25787767

[10] Loi S, Dushyanthen S, Beavis PA, Salgado R, Denkert C, Savas P (2016). RAS/MAPK activation is associated with reduced tumor-infiltrating lymphocytes in triple-negative breast cancer: therapeutic cooperation between MEK and PD-1/PD-L1 immune checkpoint inhibitors. Clin Cancer Res.

[11] Boni A, Cogdill AP, Dang P, Udayakumar D, Njauw CN, Sloss CM (2010). Selective BRAFV600E inhibition enhances T-cell recognition of melanoma without affecting lymphocyte function. Cancer Res.

[12] Kono M, Dunn IS, Durda PJ, Butera D, Rose LB, Haggerty TJ (2006). Role of the mitogen-activated protein kinase signaling pathway in the regulation of human melanocytic antigen expression. Mol Cancer Res.

[13] Brea EJ, Oh CY, Manchado E, Budhu S, Gejman RS, Mo G (2016). Kinase regulation of human MHC class I molecule expression on cancer cells. Cancer Immunol Res..

[14] Mimura K, Shiraishi K, Mueller A, Izawa S, Kua LF, So J (2014). The MAPK pathway is a predominant regulator of HLA-A expression in esophageal and gastric cancer. J Immunol.

[15] Allegrezza MJ, Rutkowski MR, Stephen TL, Svoronos N, Perales-Puchalt A, Nguyen JM (2016). Trametinib drives T-cell-dependent control of KRAS-mutated tumors by inhibiting pathological Myelopoiesis. Cancer Res.

[16] Kvistborg P, Philips D, Kelderman S, Hageman L, Ottensmeier C, Joseph-Pietras D, et al. Anti-CTLA-4 therapy broadens the melanoma-reactive CD8+ T cell response*.* Sci Transl Med. 2014; 6(254):254ra128. 10.1126/scitranslmed.3008918 25232180

[17] Ribas A, Hanson DC, Noe DA, Millham R, Guyot DJ, Bernstein SH (2007). Tremelimumab (CP-675,206), a cytotoxic T lymphocyte associated antigen 4 blocking monoclonal antibody in clinical development for patients with cancer. Oncologist.

[18] Kortum RL, Rouquette-Jazdanian AK, Samelson LE (2013). Ras and extracellular signal-regulated kinase signaling in thymocytes and T cells. Trends Immunol.

[19] Castle JC, Loewer M, Boegel S, de Graaf J, Bender C, Tadmor AD (2014). Immunomic, genomic and transcriptomic characterization of CT26 colorectal carcinoma. BMC Genomics.

[20] Davies BR, Logie A, McKay JS, Martin P, Steele S, Jenkins R (2007). AZD6244 (ARRY-142886), a potent inhibitor of mitogen-activated protein kinase/extracellular signal-regulated kinase kinase 1/2 kinases: mechanism of action in vivo, pharmacokinetic/pharmacodynamic relationship, and potential for combination in preclinical models. Mol Cancer Ther.

[21] Crane MJ, Daley JM, van Houtte O, Brancato SK, Henry WL, Albina JE (2014). The monocyte to macrophage transition in the murine sterile wound. PLoS One.

[22] Laoui D, Van Overmeire E, Di Conza G, Aldeni C, Keirsse J, Morias Y (2014). Tumor hypoxia does not drive differentiation of tumor-associated macrophages but rather fine-tunes the M2-like macrophage population. Cancer Res.

[23] Ostuni R, Kratochvill F, Murray PJ, Natoli G (2015). Macrophages and cancer: from mechanisms to therapeutic implications. Trends Immunol.

[24] Van Overmeire E, Stijlemans B, Heymann F, Keirsse J, Morias Y, Elkrim Y (2016). M-CSF and GM-CSF receptor signaling differentially regulate Monocyte maturation and macrophage polarization in the tumor microenvironment. Cancer Res.

[25] Tosolini M, Kirilovsky A, Mlecnik B, Fredriksen T, Mauger S, Bindea G (2011). Clinical impact of different classes of infiltrating T cytotoxic and helper cells (Th1, th2, treg, th17) in patients with colorectal cancer. Cancer Res.

[26] Rodriguez PC, Ochoa AC (2008). Arginine regulation by myeloid derived suppressor cells and tolerance in cancer: mechanisms and therapeutic perspectives. Immunol Rev.

[27] Youn JI, Nagaraj S, Collazo M, Gabrilovich DI (2008). Subsets of myeloid-derived suppressor cells in tumor-bearing mice. J Immunol.

[28] D'Souza WN, Chang CF, Fischer AM, Li M, Hedrick SM (2008). The Erk2 MAPK regulates CD8 T cell proliferation and survival. J Immunol.

[29] D'Souza WN, Lefrancois L (2003). IL-2 is not required for the initiation of CD8 T cell cycling but sustains expansion. J Immunol.

[30] Selby MJ, Engelhardt JJ, Quigley M, Henning KA, Chen T, Srinivasan M (2013). Anti-CTLA-4 antibodies of IgG2a isotype enhance antitumor activity through reduction of intratumoral regulatory T cells. Cancer Immunol Res.

[31] Gabrilovich DI, Ostrand-Rosenberg S, Bronte V (2012). Coordinated regulation of myeloid cells by tumours. Nat Rev Immunol.

[32] Ugel S, De Sanctis F, Mandruzzato S, Bronte V (2015). Tumor-induced myeloid deviation: when myeloid-derived suppressor cells meet tumor-associated macrophages. J Clin Invest.

[33] Sinha P, Clements VK, Fulton AM, Ostrand-Rosenberg S (2007). Prostaglandin E2 promotes tumor progression by inducing myeloid-derived suppressor cells. Cancer Res.

[34] Zelenay S, van der Veen AG, Bottcher JP, Snelgrove KJ, Rogers N, Acton SE (2015). Cyclooxygenase-dependent tumor growth through evasion of immunity. Cell.

[35] Janne PA, van den Heuvel MM, Barlesi F, Cobo M, Mazieres J, Crino L (2017). Selumetinib plus Docetaxel compared with Docetaxel alone and progression-free survival in patients with KRAS-mutant advanced non-small cell lung cancer: the SELECT-1 randomized clinical trial. JAMA.

[36] Ribas A, Butler M, Lutzky J, Lawrence DP, Robert C, Miller W (2015). Phase I study combining anti-PD-L1 (MEDI4736) with BRAF (dabrafenib) and/or MEK (trametinib) inhibitors in advanced melanoma. J Clin Oncol.

[37] Bendell JC, Kim TW, Goh BC, Wallin J, Oh D-Y, Han S-W (2016). Clinical activity and safety of cobimetinib (cobi) and atezolizumab in colorectal cancer (CRC). J Clin Oncol.

