# Supplementary material for: The MEK inhibitor selumetinib complements CTLA-4 blockade by reprogramming the tumor immune microenvironment
Source: J Immunother Cancer. 2017 Aug 15;5:63. doi: 10.1186/s40425-017-0268-8 (PMC5557252; doi:10.1186/s40425-017-0268-8)
Supplement: Supplementary file 2 — Figure S1. Selumetinib alters the phenotype of LPS-activated human monocyte-derived dendritic cells in vitro. Figure S2. Primary immune response to KLH immunization in vivo with or without concurrent selumetinib, anti-CTLA-4 or combination treatment. Figure S3. Viability of CT26 mouse tumor cell line exposed to selumetinib in vitro. Figure S4. Numbers of splenic and intratumoral T-cell subsets following selumetinib, anti-CTLA-4 and combination treatment in vivo. (PPTX 906 kb) [file 40425_2017_268_MOESM2_ESM.pptx]

## Slide 1
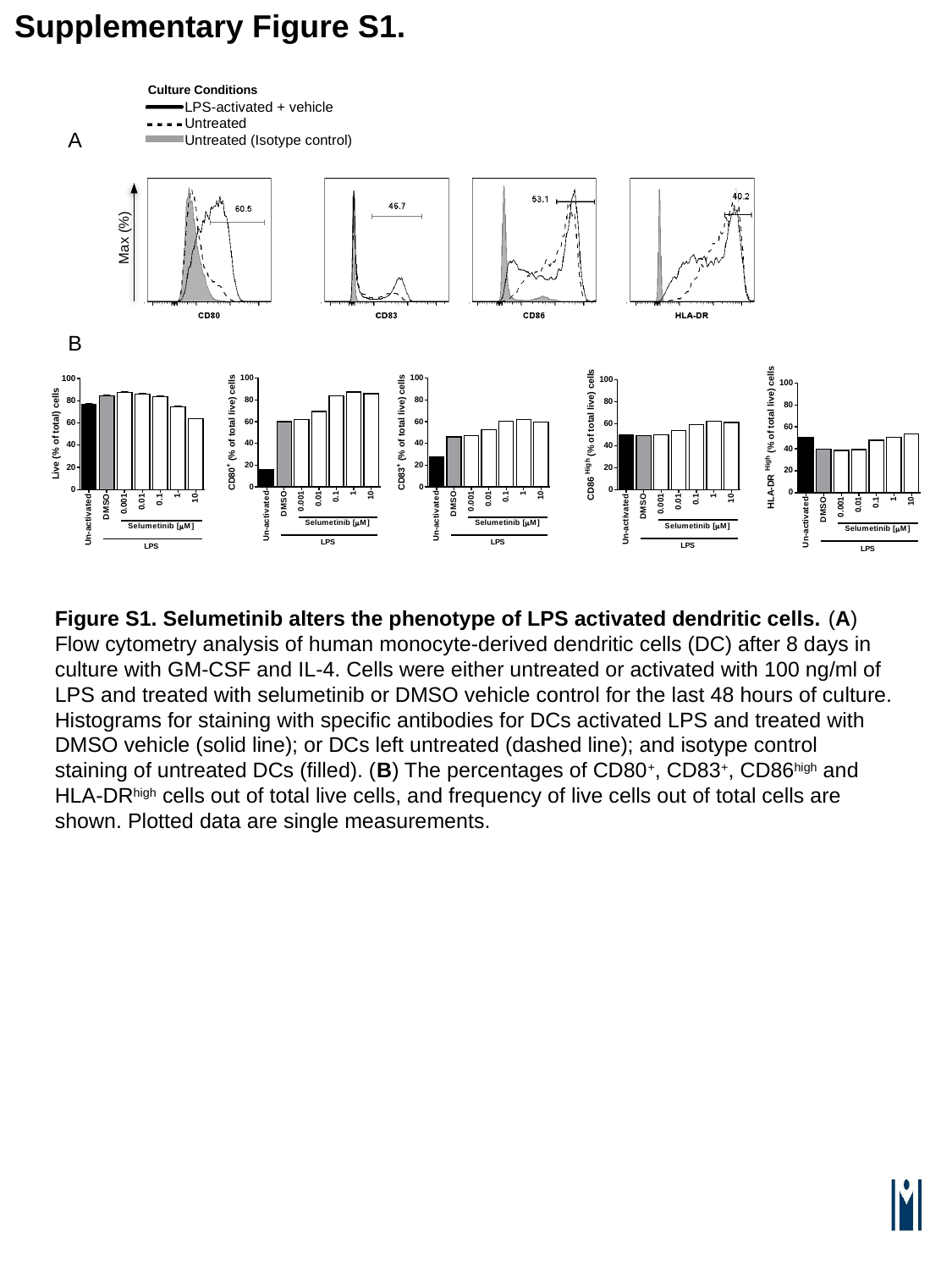

Supplementary Figure S1.
Culture Conditions
LPS-activated + vehicle
Untreated
Untreated (Isotype control)
A
Max (%)
B
Figure S1. Selumetinib alters the phenotype of LPS activated dendritic cells. (A) Flow cytometry analysis of human monocyte-derived dendritic cells (DC) after 8 days in culture with GM-CSF and IL-4. Cells were either untreated or activated with 100 ng/ml of LPS and treated with selumetinib or DMSO vehicle control for the last 48 hours of culture. Histograms for staining with specific antibodies for DCs activated LPS and treated with DMSO vehicle (solid line); or DCs left untreated (dashed line); and isotype control staining of untreated DCs (filled). (B) The percentages of CD80+, CD83+, CD86high and HLA-DRhigh cells out of total live cells, and frequency of live cells out of total cells are shown. Plotted data are single measurements.

## Slide 2
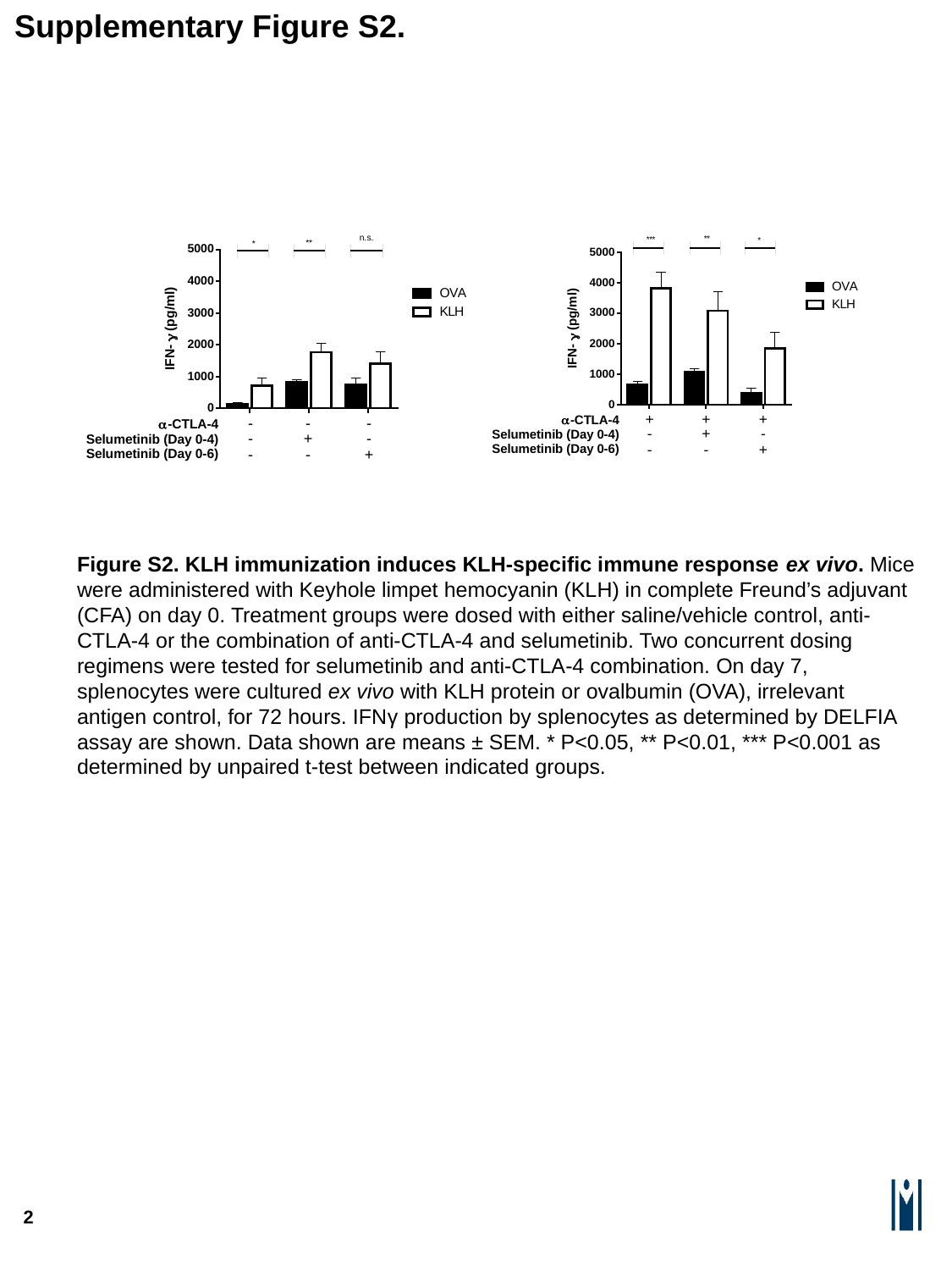

Supplementary Figure S2.
Figure S2. KLH immunization induces KLH-specific immune response ex vivo. Mice were administered with Keyhole limpet hemocyanin (KLH) in complete Freund’s adjuvant (CFA) on day 0. Treatment groups were dosed with either saline/vehicle control, anti-CTLA-4 or the combination of anti-CTLA-4 and selumetinib. Two concurrent dosing regimens were tested for selumetinib and anti-CTLA-4 combination. On day 7, splenocytes were cultured ex vivo with KLH protein or ovalbumin (OVA), irrelevant antigen control, for 72 hours. IFNγ production by splenocytes as determined by DELFIA assay are shown. Data shown are means ± SEM. * P<0.05, ** P<0.01, *** P<0.001 as determined by unpaired t-test between indicated groups.
2

## Slide 3
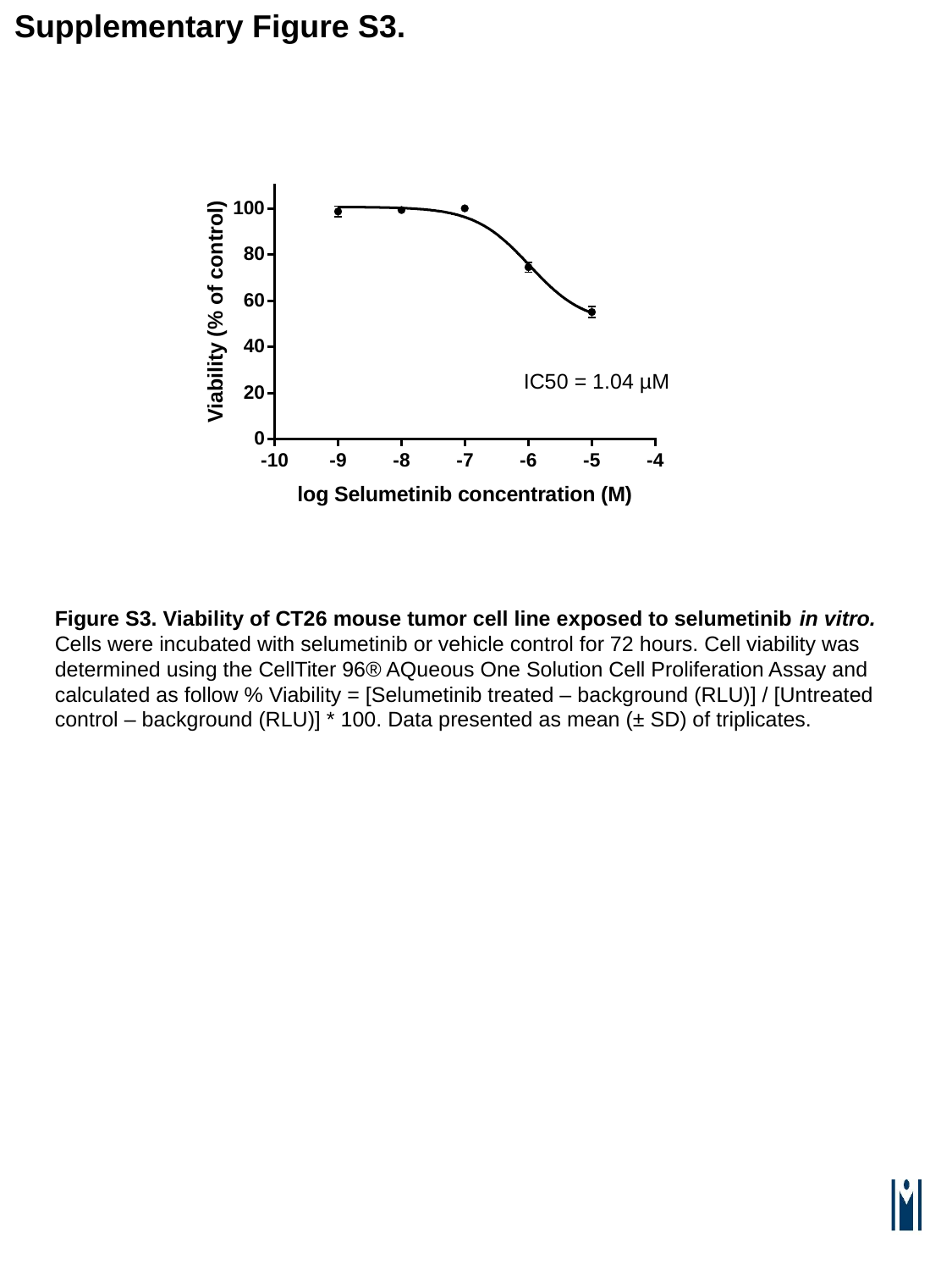

Supplementary Figure S3.
IC50 = 1.04 µM
Figure S3. Viability of CT26 mouse tumor cell line exposed to selumetinib in vitro. Cells were incubated with selumetinib or vehicle control for 72 hours. Cell viability was determined using the CellTiter 96® AQueous One Solution Cell Proliferation Assay and calculated as follow % Viability = [Selumetinib treated – background (RLU)] / [Untreated control – background (RLU)] * 100. Data presented as mean (± SD) of triplicates.

## Slide 4
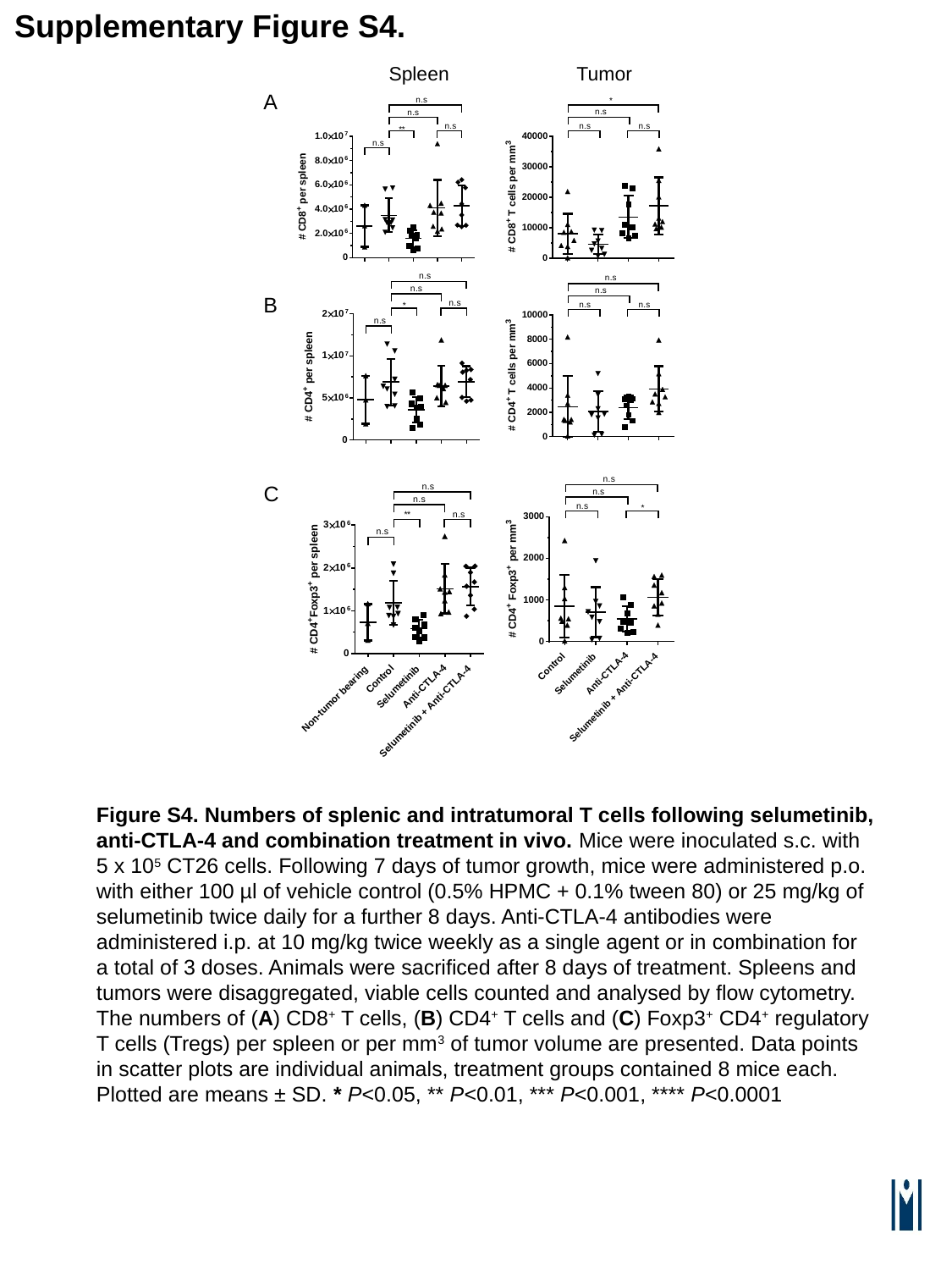

Supplementary Figure S4.
Spleen
Tumor
A
B
C
Figure S4. Numbers of splenic and intratumoral T cells following selumetinib, anti-CTLA-4 and combination treatment in vivo. Mice were inoculated s.c. with 5 x 10^5^ CT26 cells. Following 7 days of tumor growth, mice were administered p.o. with either 100 µl of vehicle control (0.5% HPMC + 0.1% tween 80) or 25 mg/kg of selumetinib twice daily for a further 8 days. Anti-CTLA-4 antibodies were administered i.p. at 10 mg/kg twice weekly as a single agent or in combination for a total of 3 doses. Animals were sacrificed after 8 days of treatment. Spleens and tumors were disaggregated, viable cells counted and analysed by flow cytometry. The numbers of (A) CD8+ T cells, (B) CD4+ T cells and (C) Foxp3+ CD4+ regulatory T cells (Tregs) per spleen or per mm3 of tumor volume are presented. Data points in scatter plots are individual animals, treatment groups contained 8 mice each. Plotted are means ± SD. * P<0.05, ** P<0.01, *** P<0.001, **** P<0.0001
